# Supplementary material for: Mixture of Synthetic Plant Volatiles Attracts More Stick Tea Thrips Dendrothrips minowai Priesner (Thysanoptera: Thripidae) and the Application as an Attractant in Tea Plantations
Source: Plants (Basel). 2024 Jul 15;13(14):1944. doi: 10.3390/plants13141944 (PMC11280358; doi:10.3390/plants13141944)
Supplement: Supplementary file 1 [file plants-13-01944-s001.zip › plants-3047951-supplementary.pdf]

Table S1. Related information of standard compounds.

Standard compounds	CAS	Purity（%）	Manufactory
p-Anisaldehyde	123-11-5	98	Sigma-Aldrich
Eugenol	97-53-0	99	Sigma-Aldrich
Farnesene, mixture of isomers	/	/	Sigma-Aldrich
3-Methyl butanal	590-86-3	97	Sigma-Aldrich
